# Violaceous, hyperkeratotic plaque in a pediatric patient

**DOI:** 10.1016/j.jdcr.2025.06.048

**Published:** 2025-07-25

**Authors:** Racquel A. Bitar, Sara O. Vargas, Amir H. Taghinia, Marilyn G. Liang

**Affiliations:** a Vascular Anomalies Center, Boston Children's Hospital, Boston, Massachusetts; b Department of Dermatology, Boston Children's Hospital, Harvard Medical School, Boston, Massachusetts; c Department of Pathology, Boston Children's Hospital, Harvard Medical School, Boston, Massachusetts; d Department of Plastic and Oral Surgery, Boston Children's Hospital, Harvard Medical School, Boston, Massachusetts

**Keywords:** child, lasers, MAP kinase kinase kinase 3, skin neoplasms

## Case description

**Fig 1:**
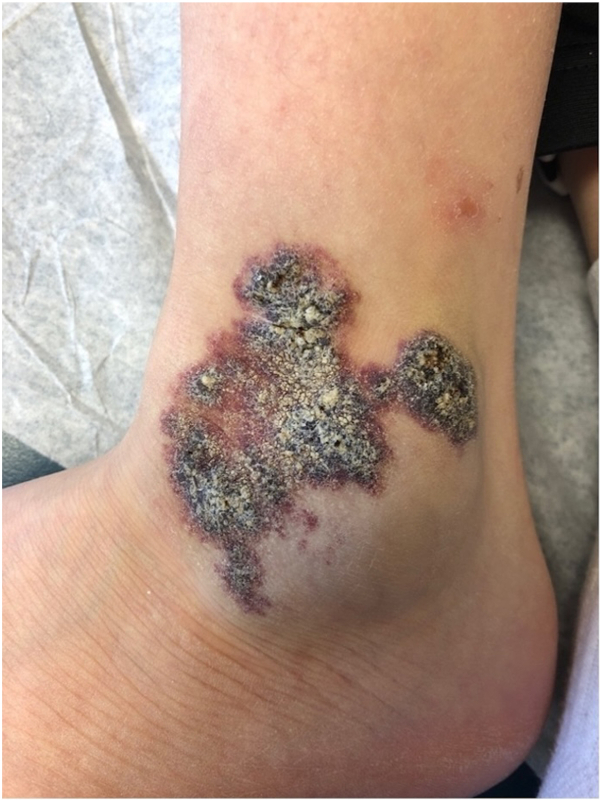
Violaceous, hyperkeratotic plaque overlying a soft, blue nodule at the left lateral malleolus.

**Fig 2:**
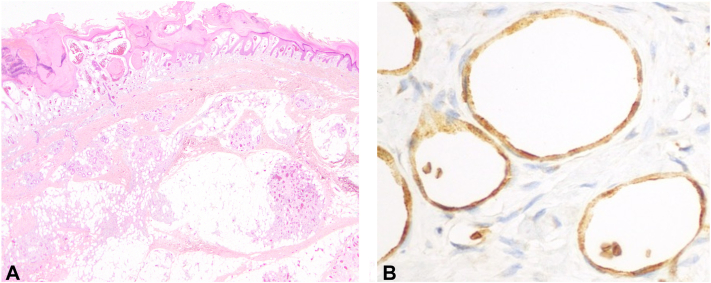
**A,** Innumerable thin-walled vascular channels, prominent in a linear pattern in the superficial dermis and as nodular collections in the subcutis. Occasional fibrin thrombi at the dermal-epidermal junction. Irregular epidermal contour, with acanthosis and parakeratosis at the surface. **B,** Immunostaining for GLUT1 (inset), highlighting both lesional endothelial cells and intraluminal red blood cells diffusely. *GLUT1*, Glucose transporter protein 1.

An 11-year-old boy presented with a painful left ankle mass, initially noted as a purple discoloration at birth. Over time, it became raised, rough, and prone to bleeding with minor trauma. CO_2_ laser treatment around 8 years of age resulted in a scar. The mass recurred at age 9. Physical examination revealed a solitary 6 × 6-centimeter, well-defined, violaceous, hyperkeratotic plaque overlying a soft, blue nodule at the left lateral malleolus (Fig 1). Excision and split-thickness skin grafting were performed for symptomatic relief. Microscopic examination revealed dermal and subcutaneous proliferation of innumerable thin-walled vascular channels, with overlying reactive epidermal changes (Fig 2, *A*). Lesional endothelium was positive for glucose transporter protein 1 (GLUT1) and negative for the lymphoendothelial marker podoplanin (D2-40) on immunohistochemical staining (Fig 2, *B*).

**Question: What is the most likely diagnosis?**

**A.** Lymphatic malformation
**B.** Angiokeratoma circumscriptum
**C.** Infantile hemangioma
**D.** Capillary-venous malformation
**E.** Verrucous venous malformation

## Discussion

Verrucous venous malformation (VVM), formerly called verrucous hemangioma, is a nonhereditary, slow-flow venous malformation that becomes progressively darker and hyperkeratotic in childhood.^1^, ^2^, ^3^ VVM is most often seen on the extremities and can be associated with pain, ulceration, or bleeding.^2^ Before it becomes hyperkeratotic, the dermoscopic vascular pattern of red to violet dots over an erythematous patch is characteristic for VVM, distinguishing it from capillary malformation or LM.^3^ Microscopic examination demonstrates hyperkeratosis, papillomatosis, and acanthosis, and proliferation of veins extending deep into the dermis and subcutaneous tissue, although deeper subcutaneous variants may lack the characteristic epidermal changes.^1^, ^2^, ^3^, ^4^ VVM harbors a somatic missense mutation in mitogen-activated protein kinase kinase kinase 3 (MAP3K3) that leads to malformed dermal venule-like channels.^4^ MAP3K3 is an enzyme downstream of the angiopoietin 1 and tunica internal endothelial cell kinase pathway; alterations in the tunica internal endothelial cell kinase pathway are associated with venous malformations.^3^^,^^4^ The molecular mechanism of MAP3K3 potentially contributing to the hyper-keratinizing appearance of VVM is unknown.

VVM is often misdiagnosed clinically, despite its unique appearance. Differential diagnoses include LM, angiokeratoma, venous malformation, capillary-LM, and IH.^5^, ^1^, ^2^, ^3^, ^4^ Size, focal GLUT1 immunopositivity, pattern of progressive growth and hyperkeratosis, and proliferation of veins in the dermis and subcutis are key diagnostic features.^1^, ^2^, ^3^, ^4^ Numerous treatments have been reported, including topical corticosteroids, radiotherapy, pulsed dye or long-pulsed neodymium-doped yttrium aluminum garnet laser therapy with excision, and oral or topical sirolimus.^2^ Several studies support that complete excision is optimal.^1^^,^^2^ The VVM presented herein illustrates the classic natural history, clinical appearance, microscopic features, and immunohistochemical profile of a distinctive, but underrecognized, pediatric condition.

## Conflicts of interest

Dr Liang is an investigator for Relay Therapeutics. Dr Vargas receives grant funding from Boehringer Ingelheim and consults for various medicolegal entities. Ms Bitar and Dr Taghinia have no conflicts of interest to declare.
